# Cost comparison of post-remission strategies in younger and older AML patients in France

**DOI:** 10.1038/s41408-023-00874-y

**Published:** 2023-06-28

**Authors:** Michael Mounie, Pierre-Yves Dumas, Sandra Liva-Yonnet, Didier Fabre, Thibault Leguay, Jean Galtier, Emilie Berard, Ramaroson Hanta, Véronique Gilleron, Sarah Bertoli, Arnaud Pigneux, Christian Récher, Nadège Costa

**Affiliations:** 1 Unité d’Evaluation Médico-Economique, Centre Hospitalier Universitaire (CHU), Toulouse, France; 2 Institut National de la Santé et de la Recherche Médicale, U1295 Toulouse, France; 3 CHU Bordeaux, Service d’Hématologie Clinique et de Thérapie Cellulaire, F-33000 Bordeaux, France; 4 Université de Bordeaux, Bordeaux, France; 5 Institut National de la Santé et de la Recherche Médicale, U1312 Bordeaux, France; 6 Département d’Information Médicale, Centre Hospitalier Universitaire (CHU), Toulouse, France; 7 Département d’Information Médicale, Centre Hospitalier Universitaire (CHU), Bordeaux, France; 8 Service d’Hématologie, Centre Hospitalier Universitaire de Toulouse, Institut Universitaire du Cancer de Toulouse Oncopole, Toulouse, France; 9 Université Toulouse III Paul Sabatier, Toulouse, France; 10 Cancer Research Center of Toulouse, Unité Mixte de Recherche (UMR) 1037 INSERM, ERL5294 Centre National de la Recherche Scientifique, Toulouse, France

**Keywords:** Chemotherapy, Acute myeloid leukaemia

Dear Editor,

The gold standard post-remission treatment in younger patients with acute myeloid leukemia (AML) in first complete remission after induction chemotherapy was based until recently on high-dose cytarabine (HDAC) delivered every 12 h on Days 1, 3, and 5 (HDAC-135) [1]. In addition, recent guidelines recommend the use of intermediate-dose cytarabine (IDAC) as higher doses were found to be largely supra-therapeutic [2, 3]. In older patients, no chemotherapy regimen has as yet demonstrated its superiority. The consolidation treatment recommended is based on IDAC similar to younger subjects [3]. These consolidation schemes have recently been challenged. Two recent studies including ours, one based on a prospective cohort study and the other based on a retrospective registry study, showed that administration of HDAC with a condensed regimen over 3 consecutive days (HDAC-123) resulted in similar rates of relapse-free survival (RFS), the cumulative incidence of relapse (CIR), non-relapse mortality (NRM) and overall survival (OS) in younger patients in addition to shorter hematological recovery times regarding neutrophils and platelets [4, 5]. On the other hand, an outpatient strategy consisting of six courses of a single-dose anthracycline combined with subcutaneous cytarabine (SDAC/a) has been developed as an alternative to IDAC in older patients, specifically by the French Innovative Leukemia Organization group [6]. We and others showed that SDAC/a led to similar rates of OS, RFS, CIR, and NRM compared to IDAC in addition to a lower rate of documented bacteremia and transfusion requirement [7–9]. Finally, both of these treatments produced a decrease in the total duration of hospitalization for the whole post-remission program and we hypothesize that this decrease leads to substantial cost reductions. In this study, we aim to formally compare the economic burden of inpatient stays during the consolidation phase between HDAC-123 and HDAC-135 as well as between SDAC/a and IDAC in younger and older patients, respectively.

This work is based on the AML French Regional Registry (DATAML), focusing on patients with newly diagnosed de novo or secondary AML according to the World Health Organization classification [10]. Younger patients were between 18 and 60 years old, and had received at least 1 cycle of HDAC as a post-remission strategy between 2008 and 2017 in the first CR or CR with incomplete hematological recovery (CRi) after 1 course of intensive induction chemotherapy. Patients received 1 to 3 cycles of HDAC 3 g/m² every 12 h for 3 days (18 g/m²) per 1 of 2 schedules: either HDAC-123 (3 g/m² every 12 h, Days 1, 2, and 3) or HDAC-135 (3 g/m² every 12 h, Days 1, 3, and 5) [5]. Older patients were ≥ 60 years old and had received at least one cycle of chemotherapy as a post-remission strategy between 2007 and 2017 in the first CR or CRi after 1 course of intensive induction chemotherapy. Patients received a post-remission schedule with 1 to 3 cycles of inpatient IDAC 1.5 g/m^2^ every 12 h for 3 days (9 g/m^2^) which was referred to as the IDAC arm; or an outpatient schedule with six courses of idarubicin 8 mg/m²/day IV on Day 1 and cytarabine 50 mg/m^2^/12 h/day subcutaneously on Days 1–5 which was referred to as the SDAC/a arm [9]. The economic analysis was performed from the French National Health Insurance (FNHI) perspective and focused on costs associated with inpatient stays. These costs correspond to diagnosis-related group (DRG) tariffs to which extra charges can be added if applicable (i.e., additional expensive medication, day prices for acute care, intensive care, or monitoring unit stays…). Periods considered were the consolidation phases similarly defined for both young and older patients: from the date of inpatient stay associated with the first cycle of consolidation to the date of the inpatient stay associated with the last cycle of consolidation plus 30 days (Supplementary Fig. 1). Further information on methods and statistics are included in Supplementary data.

Of the 240 younger patients recruited, 224 were analyzable, and 83 and 141 patients with HDAC-123 and HDAC-135 respectively. Both groups had similar clinical characteristics except for a higher proportion of males in the HDAC-123 arm (Table 1). Treatment period durations and number of cycles were also similar between groups. Five patients in the HDAC-123 group received an allogeneic HSCT during the study period while 4 patients in the HDAC-135 group experienced a relapse. The total costs for HDAC-123 and HDAC-135 were €36,818 and €39,805 respectively within the considered period (Supplementary Table 1). When adjusting for confoundable factors, a significant 16% cost reduction (RR = 0.84, CI: [0.75–0.94] *p* = 0.0016) in favor of HDAC-123 was found (Table 2).

**Table 1:** Description of younger and older AML populations according to post-remission treatment arms.

		AML patients 18–60 y			AML patients > 60 y		
		HDAC-123	HDAC-135	*p*	SDAC	IDAC	*p*
		*n* = 83	*n* = 141		*n* = 271	*n* = 69	
Center, *n* (%)	Toulouse	36 (43.4)	87 (71.7)	0.009	143 (93.5)	10 (6.5)	<0.001
	Bordeaux	47 (56.6)	54 (38.3)		128 (68.5)	59 (31.5)	
Age, mean (sd)		43.9 (11.2)	45.1 (11.7)	0.442	68.6 (4.9)	64.9 (3.2)	<0.001
Sex, *n* male (%)		52 (62.7)	68 (48.2)	0.039	169 (62,2)	39 (56)	0.408
Cytogenetic risk, *n* (%)	Favorable	30 (36)	37 (26.4)	0.246	9 (3.3)	12 (17.4)	<0.001
	Intermediate	43 (51.8)	88 (62.4)		232 (85.6)	34 (49.3)	
	Adverse	10 (12)	16 (11.3)		30 (11.1)	23 (33.3)	
N post-remissions session, *n* (%)	1	11 (13)	22 (15.6)	0.281	47 (17.3)	8 (11.6)	<0.001
	2	31 (37.3)	38 (27)		48 (17.7)	23 33.3)	
	3	41 (49.4)	81 (57.4)		24 (8.9)	38 (55.1)	
	≥4	0	0		158 (58.3)	0	
Period length (days), mean (sd)		85 (32)	89 (35)	0.357	159,1 (104)	93,5 (50)	<0.001
Allo-HSCT, *n* (%)		5 (6)	0	0.006	4 (1,48)	2 (2,9)	0.352
Relapse or death during the study period, *n* (%)		0	4 (5,6)	0.299	17 (6.3)	2 (2.9)	0.385

*AML* acute myeloid leukemia, *HDAC-123* high-dose cytarabine on Days 1–3, *HDAC-135* high-dose cytarabine on Days 1, 3, and 5, *SDAC* standard doses of cytarabine associated with a dose of anthracycline, *IDAC* Intermediate doses of cytarabine, *CI* confidence interval.

**Table 2:** Adjusted effect of post-remission treatment on cost according to younger and older AML population.

		AML patients ≥18 and ≤60 years old		AML patients >60 years old	
Variables		Exp(*β*) [95 CI]	*p*	Exp(*β*) [95 CI]	*p*
Age		1 [1–1.01]	0.427	1.01 [0.98–1.03]	0.628
Sex	Women	1		1	
	Men	1.01 [0.91–1.13]	0.802	0.95 [0.74–1.23]	0.722
Allo-HSCT	0	1		1	
	1	3.28 [2.86–3.77]	<0.001	2.00 [1.18–3.38]	0.010
	Favorable	1		1	
Cytogenetic risk	Intermediate	1.02 [0.85–1.23]	0.826	1.11 [0.82–1.49]	0.584
	Adverse	1.06 [0.98–1.16]	0.162	0.70 [0.46–1.06]	0.090
Relapse or death during study period	0	1		1	
	1	0.99 [0.79–1.24]	0.932	1.23 [0.59–2.57]	0.584
Treatment for younger AML	HDAC-135	1			
	HDAC-123	0.84 [0.75–0.94]	0.002		
Numbers of cycles		1.57 [1.45–1.7]	<0.001		
Treatment for older AML	IDAC			1	
	SDAC/a			0.24 [0.18–0.31]	<0.001

Generalized estimating equation model with Gamma distribution and log link; *AML* acute myeloid leukemia, *HDAC-123* high-dose cytarabine on Days 1–3, *HDAC-135* high-dose cytarabine on Days 1, 3, and 5, *SDAC* standard doses of cytarabine associated with a dose of anthracycline, *IDAC* intermediate doses of cytarabine, *CI* confidence interval.

Of the 395 older patients, 340 were analyzable, and 271 and 69 patients with SDAC/a and IDAC, respectively (Table 1). Patients in the SDAC/a group were significantly older than patients treated with IDAC and had a higher frequency of intermediate cytogenetic risk. Finally, SDAC/a treatment required 4 or more cycles for 58% of patients leading to a significantly longer period considered (159.1 days) in comparison with IDAC (93.5 days). The total costs for SDAC/a and IDAC within the period considered were €11,763 and €40,253, respectively (Supplementary Table 1). For older patients, the cost decrease observed in the bivariate analysis was further confirmed by multivariate analysis where a 76% (RR = 0.24; CI: [0.18–0.31]; *p* < 0.001) reduction in costs was revealed for the SDAC/a group (Table 2).

We have completed the cost comparison of two post-remission treatments for younger (HDAC-123) and older (SDAC/a) AML patients versus standards of care and considering inpatient stay costs using the FNHI perspective. When applied to the HDAC-135 related management cost of €39,805, a 16% cost decrease represents an average €6,000 cost saving per patient in the HDAC-123 arm, and when applied to the IDAC related management cost of €40,253, a 76% cost decrease represents an average €30,000 cost saving per patient in the SDAC/a arm. In both comparisons, the cost reduction is mainly due to a significant decrease in the length of inpatient stay and therefore less expensive inpatient stays (Supplementary Fig. 2). The cost reduction may be underestimated when considering the hospital perspective. DRG tariffs include hospital charges during a defined range of days. As an example, the duration for an inpatient stay with DRG code generally used for consolidation cycles (i.e., 17M051—Chemotherapy for acute leukemia, level 1) comprises between 0 and 7 days and so, similar DRG tariffs are generally applied for both HDAC-123 (3 days) or HDAC-135 (5 days). On the other hand, care management at home may increase outpatient as well as informal care costs and may diminish the cost reduction estimated if a broader perspective is considered. Moreover, it should be noted that outpatient care in lieu of inpatient care generally leads to increased quality of life and may make SDAC/a cost-effective [11]. In France, cancer management is 100% reimbursed by the FNHI, no matter inpatient or outpatient care. In this context, the FNHI may save costs nevertheless the hospital which uses activity-based pricing, may lose funding.

Our study suffers from some limitations. The main one relates to its retrospective nature and the heterogeneity between groups compared in terms of patient characteristics in addition to population distribution between groups. Multivariate analyses have notably been implemented to adjust for confounding factors to take into account these differences. In addition, we were only able to consider inpatient hospital stays at the university hospital level, leading to the exclusion of patients without all consolidation-related inpatient stays traced in the database. Furthermore, all inpatient stays in periphery hospitals were not considered, although according to AML management settings, we believe they remain sparse. We were also not able to take into account outpatient management in our analysis in addition to informal care costs as the data were unavailable in the database used. In this context, the incremental cost between SDAC/a, mainly administered on an outpatient basis and IDAC may be overestimated when considering a larger economic perspective. Finally, our data focused on two hospital centers but these post-remission strategies match worldwide standards.

Much remains to be done to improve AML post-remission treatment notably in terms of cost saving. According to AML incidence in France and taking into account patient age distribution and the fact that 50% of patients have chemotherapy and 70% of them undergo post-remission treatment, 1200 AML patients made up of 317 younger and 883 older subjects respectively receive post-remission regimens annually [12]. In accordance with our results, we may crudely extrapolate an annual 28-million-euro cost saving due to HDAC-123 and SDAC/a in France within the FNHI (i.e., 1.9 million and 26.5 million euros for younger and older patients, respectively).

We have shown that substantial cost savings are attainable during the consolidation treatment phase by using HDAC-123 and SDAC/a. Condensed regimens and outpatient management produce a drastic decrease in the average length of stay which leads to an estimated cost reduction. In addition, this may lessen the perceived burden of AML management and free up space in hospitals. Although HDAC-123 and SDAC/a are not definitively clinically superior to gold standard alternatives, we cannot put out of sight the meaningful cost savings resulting from these management regimens. Thus, this study provides important information for patients, hematologists who treat AML, health politics, and funding of clinical research.

## Supplementary information

Supplementary files document

## References

[1] Mayer RJ, Davis RB, Schiffer CA, Berg DT, Powell BL, Schulman P (1994). Intensive postremission chemotherapy in adults with acute myeloid leukemia. N Engl J Med.

[2] Löwenberg B (2013). Sense and nonsense of high-dose cytarabine for acute myeloid leukemia. Blood.

[3] Döhner H, Wei AH, Appelbaum FR, Craddock C, DiNardo CD, Dombret H (2022). Diagnosis and management of AML in adults: 2022 recommendations from an international expert panel on behalf of the ELN. Blood.

[4] Jaramillo S, Benner A, Krauter J, Martin H, Kindler T, Bentz M (2017). Condensed versus standard schedule of high-dose cytarabine consolidation therapy with pegfilgrastim growth factor support in acute myeloid leukemia. Blood Cancer J.

[5] Dumas PY, Bertoli S, Bérard E, Leguay T, Tavitian S, Galtier J (2020). Delivering HDAC over 3 or 5 days as consolidation in AML impacts health care resource consumption but not outcome. Blood Adv..

[6] Pigneux A, Béné MC, Salmi LR, Dumas PY, Delaunay J, Bonmati C (2018). Improved survival by adding lomustine to conventional chemotherapy for elderly patients with AML without unfavorable cytogenetics: results of the LAM-SA 2007 FILO Trial. J Clin Oncol.

[7] Itzykson R, Gardin C, Pautas C, Thomas X, Turlure P, Raffoux E (2011). Impact of post-remission therapy in patients aged 65–70 years with de novo acute myeloid leukemia: a comparison of two concomitant randomized ALFA trials with overlapping age inclusion criteria. Haematologica.

[8] Gardin C, Turlure P, Fagot T, Thomas X, Terre C, Contentin N (2007). Postremission treatment of elderly patients with acute myeloid leukemia in first complete remission after intensive induction chemotherapy: results of the multicenter randomized Acute Leukemia French Association (ALFA) 9803 trial. Blood.

[9] Galtier J, Alric C, Bérard E, Leguay T, Tavitian S, Bidet A (2021). Intermediate-dose cytarabine or standard-dose cytarabine plus single-dose anthracycline as post-remission therapy in older patients with acute myeloid leukemia: impact on health care resource consumption and outcomes. Blood Cancer J.

[10] Arber DA, Orazi A, Hasserjian R, Thiele J, Borowitz MJ, Le Beau MM (2016). The 2016 revision to the World Health Organization classification of myeloid neoplasms and acute leukemia. Blood.

[11] Hinz A, Weis J, Faller H, Brähler E, Härter M, Keller M (2018). Quality of life in cancer patients-a comparison of inpatient, outpatient, and rehabilitation settings. Support Care Cancer.

[12] Defossez G, Le Guyader-Peyrou S, Uhry Z, Grosclaude P, Colonna M, Dantony E, et al. National estimates of cancer incidence and mortality in metropolitan France between 1990 and 2018. Overview. Saint-Maurice: Santé Publique France, 2019.

