# Supplementary material for: Cost comparison of post-remission strategies in younger and older AML patients in France
Source: Blood Cancer J. 2023 Jun 28;13(1):100. doi: 10.1038/s41408-023-00874-y (PMC10307787; doi:10.1038/s41408-023-00874-y)
Supplement: Supplementary file 1 — Supplementary files document [file 41408_2023_874_MOESM1_ESM.docx]

Supplementary figure 1. AML patients consolidation phases depending on treatment


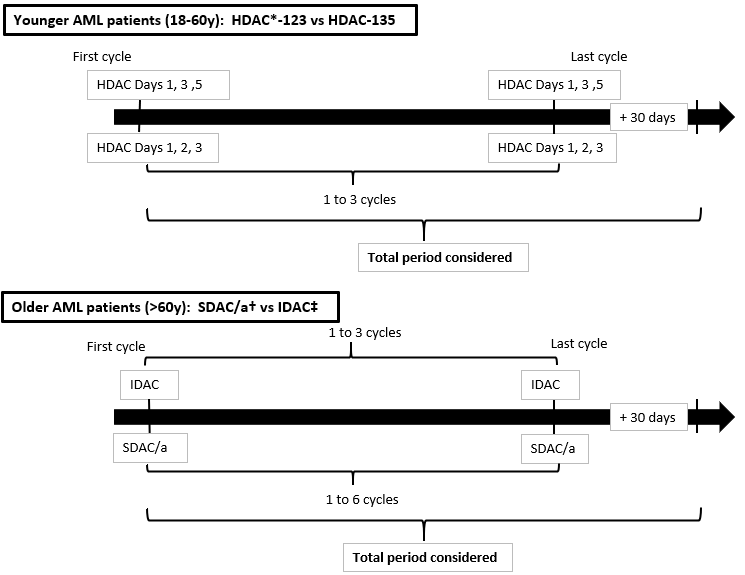


Supplementary methods. **Detailed materials and methods**

*Study design and patients*

This retrospective study is based on the AML French Regional Registry (DATAML) for Toulouse and Bordeaux in France. For both younger and older cohorts, patients had newly diagnosed de novo or secondary AML according to the World Health Organization 2016 classification, excluding acute promyelocytic leukemia.

Younger patients were included in this study if they were between 18 and 60 years old, had received at least 1 cycle of HDAC as post-remission strategy between January 1, 2008 and June 30, 2017 in first CR or CR with incomplete hematological recovery (CRi) after 1 course of intensive induction chemotherapy. Patients received 1 to 3 cycles of HDAC 3 g/m² every 12 hours for 3 days (18 g/m²) per 1 of 2 schedules: either HDAC-123 (3 g/m² every 12 hours, Days 1, 2, and 3) or HDAC-135 (3 g/m² every 12 hours, Days 1, 3, and 5) (Supplementary file 3).

Older patients were included in this study if they were ≥ 60 years old, had received at least one cycle of chemotherapy as post-remission strategy between January 1, 2007 and May 31, 2017 in first CR or CRi after 1 course of intensive induction chemotherapy. Patients received post- remission schedule with one to three cycles of inpatient IDAC 1.5 g/m^2^ every 12 hours for 3 days (9 g/m^2^) which was referred to as the IDAC arm; or an outpatient schedule with six courses of idarubicin 8 mg/m²/day IV on Day 1 and cytarabine 50 mg/m²/12 hours/day subcutaneously on Days 1 to 5 which was referred to as the SDAC/a arm (Supplementary file 3).

The study was conducted in accordance with the Declaration of Helsinki, allowing the collection of clinical data from the anonymized French Toulouse-Bordeaux DATAML Registry.

*Economic analysis*

The economic analysis was performed from the French National Health Insurance (FNHI) perspective and focused on costs associated with inpatient stays. In France, public and private hospital fees are based on diagnosis-related group (DRG) tariffs to which extra charges can be added if applicable (i.e. additional expensive medication such as hospital-reserved drugs with special constraints, day prices for acute care, intensive care or monitoring unit stays…). DRG costs include medical and paramedical staff fees, structural costs and common drugs but do not allow access to these details. The French Health Insurance scheme, through DRG tariffs, fully reimburses the cost of the inpatient stay to the hospital except for the daily fee of 20 euros which remains the responsibility of patients. DRG tariffs also include the price of medication not counted as additional expensive medicines. In our context, post-remission chemotherapy prices are included in DRG tariffs. Inpatient Discharge Hospital Databases were used which meant that only inpatient stays taking place at Toulouse and Bordeaux University Hospital were considered. Exact dates of consolidation cycles were available in the clinical database and allowed the correct identification of related inpatient stays. We were not able to consider inpatient stays taking place in peripheral hospitals. Therefore, only patients with consolidation cycles related to inpatient stays found in the Inpatient Database were considered in the analyses explaining disparities in the sample size in comparison with previously published studies.

Consolidation phases were similarly defined for both young and older patients: from the date of inpatient stay associated with the first cycle of consolidation to the date of the inpatient stay associated with the last cycle of consolidation plus 30 days (Supplementary file 4). The 30 days were chosen to allow for aplasia and the need for re-hospitalization after the last cycle of consolidation as well as to avoid taking into account Allo-HSCT management which generally occurs after this period.

We have used the DRG tariffs directly available in the Inpatient Database ranging from 2008 to 2017 and 2007 to 2017 for younger and older patient respectively. Additionally, intensive care, continuous monitoring, acute care and expensive drug costs were taken into account. Costs were inflated to €2020 using French annual consumer price indices to take into account inflation from one year to the next in order to be comparable with the literature.

In addition to the inpatient stay costs related to management, the total number of inpatient stays and the total length of stay in days were assessed. In this context, ambulatory inpatient care counts as one inpatient stay but zero hospital day. Cytogenetic risk classification was defined according to the UK MRC classification and comorbidity index was defined according to the hematopoietic cell transplantation specific comorbidity index.

*Statistical analyses*

Descriptive statistics were implemented to summarize the data using mean ± standard deviation or occurrence with percentage. Student or Fisher two-sided tests were used to compare patient characteristics between group characteristics, HDAC-123 vs HDAC-135 and SDAC/vs IDAC respectively for younger and older patients. Similar analyses were carried out for the younger and older patients.

Hospitalization cost management was described using mean and bias-corrected as well as accelerated bootstrap 95% confidence intervals (CI). Mann Whitney Wilcoxon non-parametric two-sided tests were implemented to compare costs between groups.

Generalized estimating equation models with Gamma distribution and log link were implemented to adjust the cost differences between groups of post-remission treatments. Age, gender, number of cycles of consolidation, if patient reached the end of period considered without disease progression (DFS) and allograft within the period were used as adjustment variables. The Charlson Comorbidities Index (CCI) was available for older patients but due to similar distributions and missing data, it was not considered in the models. The number of consolidation cycles was only used in the HDAC-123 vs HDAC-135 comparison given SDAC requires more consolidation cycles than IDAC.

Statistical analyses were implemented using the R software, version 4.0.3.

**Data Availability**

Due to the nature of this research, coming from claim database merged with registry, we cannot share individual’s data publicly. Upon acceptable request, we can investigate and share aggregate data (Send requests via e-mail to the corresponding author).

Supplementary table 1. Unadjusted inpatient costs of younger and older AML populations according to post-remission treatment arms.

|  | AML patients 18-60y | | | AML patients >60y | | |
| --- | --- | --- | --- | --- | --- | --- |
| Cost (€2020) | HDAC-123 | HDAC-135 | P-value | SDAC/a | IDAC | P-value |
|  | Mean  [95% IC] | Mean  [95% IC] |  | Mean  [95% IC] | Mean  [95% IC] |  |
| Medicine, Surgery, Obstetrics | 28782  [25834; 32711] | 32282  [29248; 35315] | 0.240 | 10421  [9209; 12181] | 33328  [29434; 37647] | <0,001 |
| Intensive care | 3756  [2726; 5102] | 4098  [3190; 5073] | 0.882 | 631  [413; 994] | 1887  [1050; 3127] | 0.234 |
| Continuous monitoring | 3342  [2614; 4142] | 2931  [2201; 3862] | 0.018 | 112  [58; 238] | 4893  [3994; 5839] | <0,001 |
| Acute care | 112  [20; 413] | 78  [24; 203] | 0.750 | 76  [13; 324] | 64  [12; 181] | 0.140 |
| Expensive drugs | 827  [404; 1464] | 417  [115; 1794] | 0.006 | 523  [391; 775] | 81  [9; 242] | <0,001 |
| **Total cost** | **36818  [32919; 41790]** | **39805  [36592; 42696]** | **0.142** | **11763  [10366; 13918]** | **40253  [35342; 45333]** | **<0,001** |
| N inpatient stays | 5  [5; 6] | 5  [5; 5] | 0.2417 | 8  [7; 8] | 6  [5; 7] | 0.009 |
| Total average inpatient length of stay (days) | 27  [25; 30] | 37  [34; 39] | <0,001 | 6  [5; 8] | 26  [24; 29] | <0,001 |
| AML: Acute myeloid leukemia; HDAC-123: High-dose cytarabine on Days 1, 2, 3; HDAC-135: High-dose cytarabine on Days 1, 3, 5; SDAC: Standard doses of cytarabine associated with a dose of anthracycline; IDAC: Intermediate doses of cytarabine; CI: Confidence interval. | | | | | | |


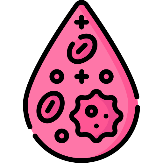

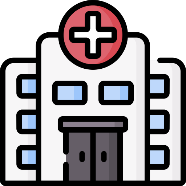
Supplementary figure 2. AML patients post-remission cycle management depending on age and treatment

Condensed **HDAC Day 123** versus **HDAC Day 135** for younger patients (18-60y) and outpatient **SDAC/a** versus **IDAC** for older patients (>60y) for consolidation treatment phase both lead to similar clinical benefits and **shorter inpatient stay lengths**


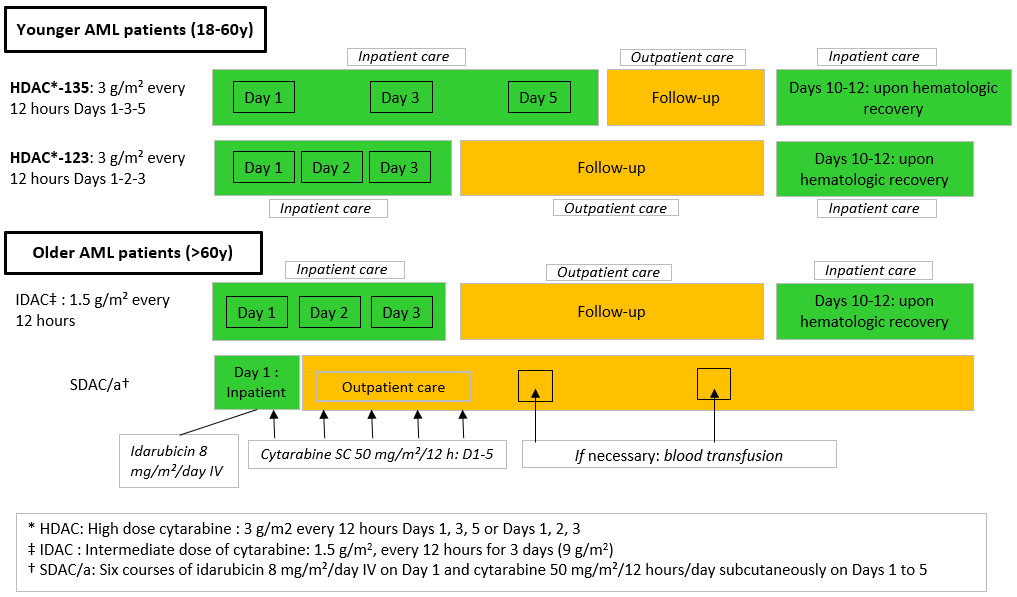


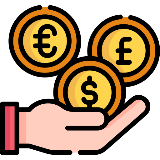

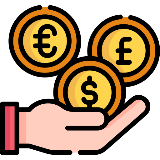


**When adjusted:**

**Substantial cost savings are attainable during the consolidation treatment phase by using HDAC-123 and SDAC/a**
